# Rapid identification of α-glucosidase inhibitors from *Poria* using spectrum-effect, component knock-out, and molecular docking technique

**DOI:** 10.3389/fnut.2023.1089829

**Published:** 2023-08-10

**Authors:** Changyang Ma, Jie Lu, Mengjie Ren, Qiuyi Wang, Changqin Li, Xuefeng Xi, Zhenhua Liu

**Affiliations:** ^1^National R&D Center for Edible Fungus Processing Technology, Henan University, Kaifeng, China; ^2^Shenzhen Research Institute of Henan University, Shenzhen, China; ^3^Joint International Research Laboratory of Food and Medicine Resource Function, Kaifeng, Henan, China; ^4^Henan Province Functional Food Engineering Technology Research Center, Kaifeng, Henan, China; ^5^College of Physical Education, Henan University, Kaifeng, Henan, China; ^6^Kaifeng Key Laboratory of Functional Components in Health Food, Kaifeng, China

**Keywords:** Poria, spectrum-effect relationships, α-glucosidase, molecular docking, rapid identification

## Abstract

**Instruction:**

Poria *(Poria cocos)* is known for its health-promoting effects and is consumed as a food due to its potential hypoglycemic activity. However, the composition of Poria is complex, and the bioactive compounds that inhibit α-glucosidase are not clear.

**Methods:**

In this study, the fingerprint of the Poria methanol extract characterized by high-performance liquid chromatography (HPLC) and the model of the corresponding spectrum-effect relationship for α-glucosidase was first established to screen the active compounds from Poria. Then, the predicted bioactive compounds were knocked out and identified using mass spectrometry. Finally, the potential binding sites and main bonds of each compound with α-glucosidase were studied using molecular docking.

**Results:**

The results have shown that at least 11 compounds from Poria could inhibit α-glucosidase effectively. Moreover, eight individual compounds, i.e., poricoic acid B **(P8)**, dehydrotumulosic acid **(P9)**, poricoic acid A **(P10)**, polyporenic acid C **(P12)**, 3- epidehydrotumulosic acid **(P13)**, dehydropachymic acid **(P14)**, 3-O-acetyl-16α-hydroxytrametenolic acid **(P21)**, and pachymic acid **(P22)**, were identified, and they exhibited effective inhibitory activity against α-glucosidase.

**Discussion:**

The possible inhibitory mechanism of them based on molecular docking showed that the binding sites are mainly found in the rings A, B, and C of these compounds, and C-3 C-16 and side chains of C-17, with the phenylalanine, arginine, tyrosine, histidine, and valine of α-glucosidase. The main interactions among them might be alkyl and hydrogen bonds, which theoretically verified the inhibitory activity of these compounds on α-glucosidase. The achievements of this study provided useful references for discovering bioactive compounds with hypoglycemic effects from Poria.

## 1. Introduction

Over the past several decades, the number of people with diabetes has increased annually and is expected to reach 693 million by 2,045 (1). α-glucosidase inhibitors, such as acarbose, voglibose, and miglitol, have been recommended as first-line hypoglycemic agents in the Asia-Pacific Diabetes Treatment Drug Guide (3rd Edition). The inhibitors can treat diabetes by inhibiting glucosidase activity in the epithelial villi of the small intestine and reducing the peak postprandial blood glucose concentration in patients (2, 3). Meanwhile, these inhibitors cause many gastrointestinal adverse effects during clinical application, such as gastric distension, diarrhea, and gastrointestinal cramping pain (4). Therefore, it is necessary to find α-glucosidase inhibitors with fewer side effects.

Many food materials with antidiabetic functions are important resources for medicine development, especially many edible fungi (5–7). According to previous research, Poria, a popular edible fungus, has been proven to have significant hypoglycemic activity (8–10) and documented in Traditional Chinese Medicine (TCM) as having clinical effectiveness in treating sugar imbalances in diabetes mellitus (11). The crude extract of Poria and its phytochemicals, such as dehydrotumulosic acid, dehydrotrametenolic acid, and pachymic acid, were proven to have insulin sensitizer activity and decrease postprandial blood glucose levels in db/db mice (12). There were a number of mechanisms explored in Poria bioactive. Dehydrotrametenolic acid could activate peroxisome proliferator-activated receptor γ (PPAR γ), which was involved with insulin resistance (13). Pachymic acid, polyporenic acid C, dehydropachymic acid, tumulosic acid, and 3-epidehydrotumulosic acid could also promote glucose uptake in 3T3-L1 adipocytes and exhibit hypoglycemic activity (14). The inhibitory effect of Poria extracts on α-glucosidase was verified in our pre-experiments. However, the composition of Poria is complex, and the corresponding bioactive compounds with inhibitive activity on α-glucosidase are still not clear.

Spectrum-effect studies are currently used as a rapid method to determine the main components with specific efficacy. In the process, the fingerprint of samples, which could reflect the type and relative contents of chemical composition in the samples, is studied using spectrum technology, while corresponding bioactivities of given samples are carried out using rapid evaluation methods. Spectrum-effect analysis can link the information of the fingerprint and biological activity through statistical methods, establish the relationship between chemical composition and the corresponding activity of the research object, identify activity-related peaks from the fingerprint, and determine the active compounds after the structure identification. The spectrum-effect relationship method has successfully been used in *Ganoderma lucidum, Malus pumila* flowers, and *Schefflera heptaphylla* for the active compounds of immunomodulation, tyrosinase inhibition, and anti-hepatoma, respectively (15–17).

**Figure 1:**
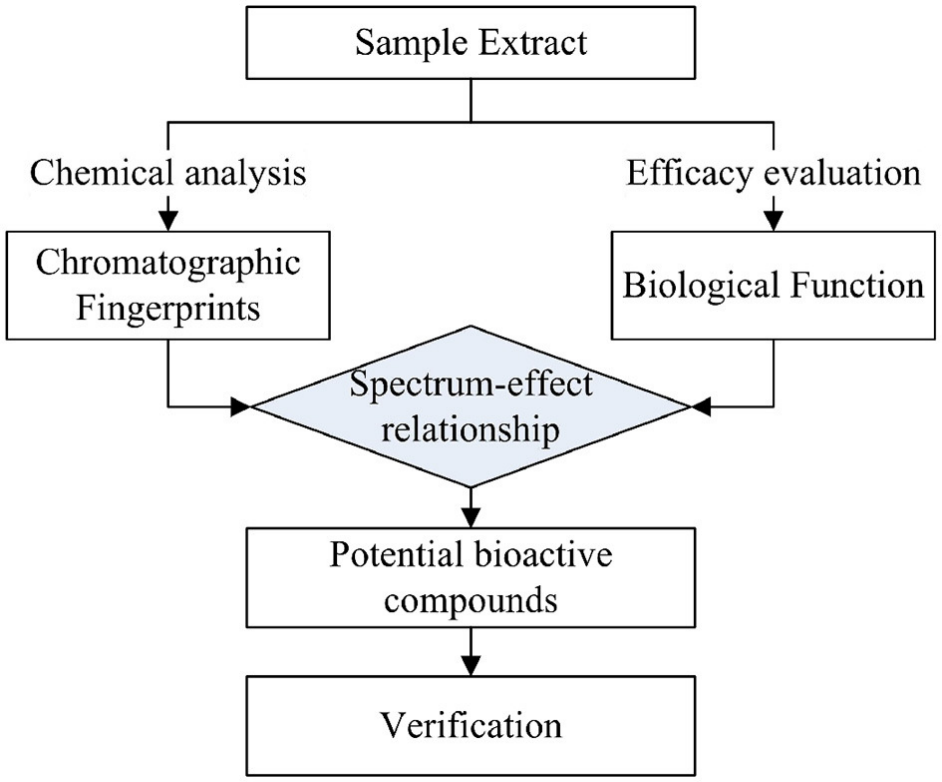
A sketch diagram of spectrum-effect relationship analysis.

Spectrum-effect relationship studies usually involve three steps (see Figure 1): First, the chromatographic fingerprints of the given samples with chemical composition information were obtained using analytical methods, and the specific efficacy of the corresponding samples was determined in *in vitro* or *in vivo* experiments (16). Then, the data on fingerprints and effects were integrated using chemometric methods to reveal the correlation between chemical composition and efficacy, thus identifying the key active substances (18). In terms of the data analysis of spectrum-effect relationships, gray correlation analysis (GRDA), partial least squares analysis (PLSR), and principal component analysis (PCA) are useful data processing methods (15, 19, 20). Among them, PLSR was more frequently used during the mechanism elucidation of TCM compared with the others, and better results were obtained (21, 22). Afterward, the predicted bioactive compounds were knocked out to verify their activities, and the actual efficacy compounds were finalized.

In this study, the spectrum-effect relationship between the chemical composition of Poria samples from different origins and their α-glucosidase inhibitory activity was carried out, and the compounds with hypoglycemic effects were identified using the knock-out technique.

## 2. Materials and methods

### 2.1. Materials

The samples of Poria were selected from seven provinces of China (Yunnan, Sichuan, Hubei, Hunan, Henan, Zhejiang, and Jiangxi). The sample authentication was performed by Professor Changqin Li at Henan University. A total of 12 batches of Poria samples were collected from different origins in 2019 without obvious different sensory characteristics, and the origin information is shown in Table 1.

**Table 1:** Different batches of Poria.

**No**.	**Origin (Province in China)**
S1	Yunnan
S2	Hubei
S3	Henan
S4	Yunnan
S5	Hunan
S6	Hunan
S7	Jiangxi
S8	Zhejiang
S9	Zhejiang
S10	Hubei
S11	Sichuan
S12	Hubei

4-Nitrophenyl-α-D-glucopyranoside (Lot: 2875129) and α-glucosidase (Lot: G5003, activity: 1 KU) were purchased from Sigma-Aldrich (Darmstadt, Germany).

An LC-20AT HPLC system (Shimadzu, Kyoto, Japan) equipped with an LC solution chromatography workstation and a Thermo BDS HYPERSIL C18 column (4.6 mm × 250 mm, 5 μm) was used for the chromatographic analysis of Poria composition. A microplate reader (Multiskan MK3) was purchased from Thermo Electron (New York, USA). A UPLC-MS/MS system equipped with a Thermo Ultimate 3000 UHPLC system and a quadrupole-exactive-orbitrap mass spectrometer was purchased from Thermo Fisher Scientific (Waltham, USA).

### 2.2. Methods

#### 2.2.1. Extraction

Each Poria sample was crushed into 80-mesh powder, and exactly 5.00 g of powder was taken for the next step. Afterward, 50 mL of methanol was added to the powder and mixed well, and then the resulting mixture was settled for 3 days with occasional shaking. The mixture was filtered, and the solid residue was extracted again two more times with the same procedure. The filtrate of three extractions was combined and concentrated in vacuo. Methanol was added to the residue to make a 1 g/mL solution, which was filtered with a 0.22 μm filter membrane prior to HPLC analysis.

#### 2.2.2. HPLC analysis

In this study, the fingerprint of Poria extract was profiled under optimized HPLC conditions by Wang (23) and Song et al. (24) as follows: flow rate was 1.4 mL/min; column temperature, 30°C; injection volume, 20 μL; and UV detection wavelength, 242 and 210 nm. The HPLC elution program was as shown in Table 2.

**Table 2:** Elution program for the fingerprint of Poria.

**t/min**	**A/% (acetonitrile)**	**B/% (0.1% phosphoric acid aqueous solution)**
0	50	50
5	55	45
12	60	40
15	65	35
19	70	30
25	75	25
30	75	25
35	100	0

#### 2.2.3. Determination method of α-glucosidase activity

According to the literature, the activity assays were performed on 96-well microtiter plates (25). The basic principle of this method was the enzymatic reaction of α-glucosidase with PNPG (4-Nitrophenyl-α-D-glucopyranoside) as substrate in the potassium phosphate buffer (PBS, pH 6.8) system. During the spectrum-effect relationship analysis, a series of concentrations of extract solution, including 1.00, 0.50, 0.25, 0.125, and 0.0625 g/mL (equivalent to the Poria sample concentration), were adopted, while 1 g/mL (Poria sample concentration) was used for evaluating the inhibitory effect of target compounds and corresponding negative solutions.

#### 2.2.4. Spectrum-effect relationship analysis

The retention time of each common peak of Poria was calibrated according to the “Chinese traditional medicine chromatographic fingerprint similarity evaluation system (2012 Edition).” Then, the peak area after equalization was taken as the independent variable (X), α-glucosidase activity rate was taken as the dependent variable (Y), and the corresponding PLSR equation was established using the analysis software DPS 7.05.

#### 2.2.5. Knock-out method for the target compounds

To obtain each compound with potential bioactivity and a corresponding negative sample, the potential target compounds predicted from 2.2.4 and the rest of the negative solution without the target fraction were collected separately according to the peak retention time of the target fraction. The details were that, after each injection of 20 μL Poria extract, all the eluate would be collected in one container after the detector except the target fractions appeared. The eluate right after the detection was collected in one container marked with the sequence number of the target fraction. For the purity of the target compound and the rest of the negative solution, only the top of the fraction peak was collected as the target compound, and the solution at the bottom of the target fraction was discarded.

After the collection of target compounds, 2.2.3 would be carried out to examine the α-glucosidase activity inhibitory rate of these target compounds and corresponding negative solutions, and the target compounds with exact bioactivity would be identified.

#### 2.2.6. Mass spectrometry analysis

Mass spectrometry is an effective method for the identification of phytochemicals (26). The compounds in the target fractions were identified using mass spectrometry analysis. The chromatographic column was a Thermo Hypersil GOLD C18 column (100 mm × 2.1 mm, 1.9 μm) with a flow rate of 0.3 mL/min. The mobile phases were 0.1% formic acid-water (A) and acetonitrile (B) with gradient elution mode as 0–2 min: 90% (A), 2–10 min: 90–95% (A), 10–13 min: 20–15% (A), 13–14 min: 5% (A), and 14–18 min: 90% (A). The injection volume was 2 μL, and the column temperature was 25°C.

#### 2.2.7. Molecular docking analysis

The protein crystal structure of α-glucosidase was obtained from the RCSB PDB protein database. According to a previous study, the protein 3D structure with high sequence similarity with α-glucosidase (α-1,4-glucosidase) from *S. cerevisiae* (PDB code 3A4A) was taken for molecular docking analysis (27, 28). In addition, the α-glucosidase used for experimental research in this paper was also the enzyme extracted from *S. cerevisiae*. The α-glucosidase macromolecule (receptor) was processed (dehydrated, hydrogenated, and charged) using Auto-Dock software (29). The npts in XYZ were set as 126, 126, and 126, the spacing value was 0.458, and the grid center was located at the XYZ coordinates (24.473, −3.89, and 16.514). Compound molecules were optimized using Chem3D 18.0 software to generate Mol2 format files.

Molecular docking of the target compounds with α-glucosidase was performed using Sybyl X2.1.1 software to assess their chemical bonding ability. The ligand molecules were mapped using the Sketch module of Sybyl X2.1.1, and the Tripos force field molecular mechanics program Minimize was used for structure optimization.

#### 2.2.8. Statistical analysis

The inhibitory bioactivity of each Poria exact and knock-out component on α-glucosidase was determined in triplicates and reported as means ± standard deviation. For multiple comparisons, a two-sided analysis of variance and a *T*-test were performed using DPS 7.05.

## 3. Results

### 3.1. The spectrum-effect analysis of Poria on α-glucosidase inhibition

#### 3.1.1. HPLC fingerprint of Poria extract

**Figure 2:**
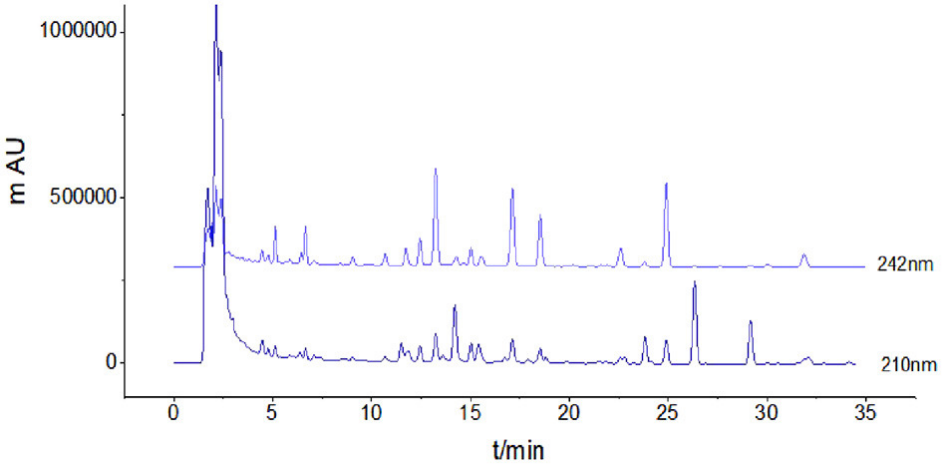
HPLC chromatogram of a Poria extract.

The HPLC chromatogram of Poria extracts at two detection wavelengths was recorded (Figure 2).

**Figure 3:**
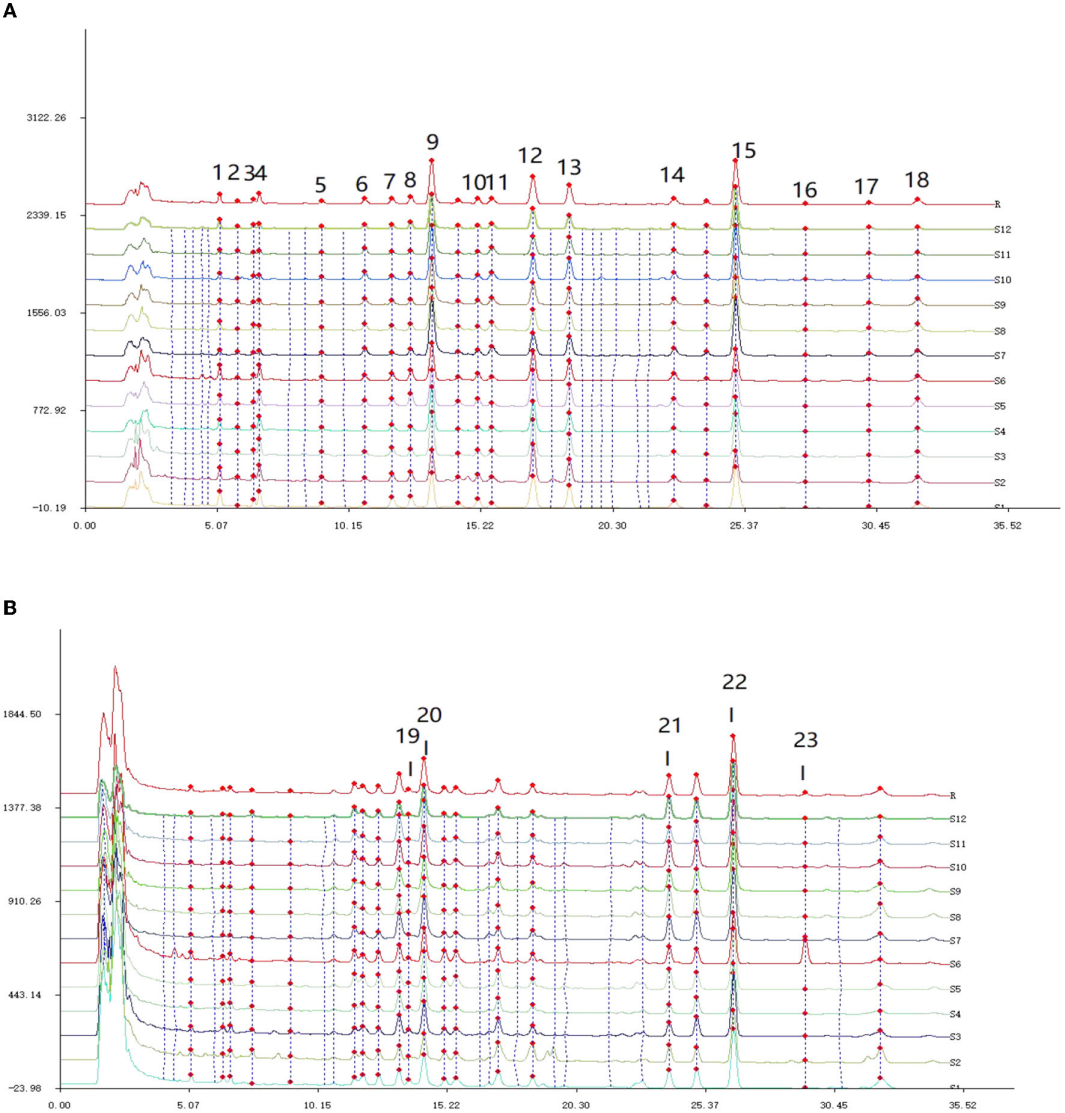
HPLC feature peak matching map of 12 batches of Poria. **(A)** The fingerprint of Poria detected at 242 nm; **(B)** the fingerprint of Poria detected at 210 nm.

The HPLC chromatogram of all batches of Poria was imported into the software “Chinese traditional medicine chromatographic fingerprint similarity evaluation system (2012 Edition)”, and the 23 common peaks with two detection wavelengths were matched in Figure 3.

From Figure 3A, it can be observed that there were a total of 18 common peaks at the detection wavelength of 242 nm, and the detection signals of **P9**, **P15**, **P12**, and **P13** were relatively high. In Figure 3B, according to corresponding retention times, five new common peaks were identified at the wavelength of 210 nm, and the detection signals of **P19**, **P20**, **P21**, and **P22** were relatively strong. Based on the 2-wavelength detection systems, a total of 23 common peaks were identified from the fingerprints of Poria.

#### 3.1.2. The α-glucosidase inhibitory effect of Poria extract

The *in vitro* α-glucosidase inhibition rates of different batches of Poria extract are shown in Table 3. The results showed that the inhibition rates of most samples at a concentration of 1 g/mL (equivalent to the raw material of Poria) ranged from 13.65 to 81.71%. Along with the decreasing concentration of Poria exacta, the corresponding inhibitory rate became weak. For multiple comparisons of the samples, the significant difference was determined with the highest inhibitory rate as the reference. For example, S6 had the strongest inhibition (81.71%) at a concentration of 1 g/mL and was significantly higher than the others (*P* < 0.001). In general, 12 batches of Poria samples varied greatly in the degree of inhibition of α-glucosidase.

**Table 3:** Inhibitory rate of α-glucosidase from 12 batches of Poria extracts ($\bar{x}$ ± s, *n* = 3).

	**The concentration of Poria exact (%)**				
**No**.	**1 g/mL**	**0.5 g/mL**	**0.25 g/mL**	**0.125 g/mL**	**0.0625 g/mL**
S1	46.05 ± 0.60^aaa^	46.13 ± 1.22^bbb^	49.67 ± 1.33	29.49 ± 1.83^ddd^	23.08 ± 1.61^eee^
S2	34.53 ± 1.61^aaa^	38.34 ± 1.53^bbb^	37.55 ± 2.39^ccc^	25.35 ± 0.98^ddd^	12.54 ± 1.73^eee^
S3	41.72 ± 0.43^aaa^	30.57 ± 1.47^bbb^	29.47 ± 2.51^ccc^	23.55 ± 1.75^ddd^	5.19 ± 1.45^eee^
S4	13.65 ± 0.93^aaa^	21.98 ± 1.79^bbb^	38.02 ± 0.33^ccc^	36.49 ± 2.19^ddd^	28.02 ± 0.08
S5	37.99 ± 1.42^aaa^	43.79 ± 0.94^bbb^	31.53 ± 2.41^ccc^	26.4 ± 1.75^ddd^	24.22 ± 1.52^eee^
S6	81.71 ± 1.45	56.21 ± 2.37	32.27 ± 1.5^ccc^	23.47 ± 1.16^ddd^	19.4 ± 1.78^eee^
S7	48.99 ± 2.74^aaa^	32.53 ± 0.89^bbb^	33.71 ± 1.92^ccc^	26.74 ± 1.9^ddd^	8.55 ± 1.36^eee^
S8	29.33 ± 2.4^aaa^	27.53 ± 2.63^bbb^	18.7 ± 1.8^ccc^	15.85 ± 2.3^ddd^	10.68 ± 2.48^eee^
S9	15.64 ± 1.04^aaa^	17.28 ± 1.37^bbb^	28.49 ± 1.2^ccc^	31.19 ± 1.1^ddd^	15.47 ± 2.87^eee^
S10	58.47 ± 0.39^aaa^	37.64 ± 2.54^bbb^	34.5 ± 1.73^ccc^	21.94 ± 1.59^ddd^	29.33 ± 1.78
S11	42.37 ± 0.87^aaa^	48.36 ± 0.89^bbb^	31.56 ± 1.85 ^ccc^	49.52 ± 2.21	26.35 ± 1.85
S12	55.37 ± 2.11^aaa^	48.36 ± 0.69^bbb^	44.2 ± 1.37^ccc^	38.24 ± 1.56^ddd^	9.32 ± 1.95^eee^

Compared at 1 g/mL with S6,

aaa *P* < 0.001; Compared at 0.5 g/mL with S6,

bbb *P* < 0.001; Compared at 0.25 g/mL with S1,

ccc *P* < 0.001; Compared at 0.125 g/mL with S11,

ddd *P* < 0.001; Compared at 0.0625 g/mL with S10,

eee *P* < 0.001.

#### 3.1.3. Spectrum-effect analysis based on PLSR

Using DPS software, the PLSR equation was developed and listed as follows:

Y = 0.000002–0.074383x1 + 0.165853x2–0.158367x3 + 0.110130x4–0.268134x5 + 0.007144x6 + 0.335275x7 + 0.592098x8–0.225877x9 + 0.076574x10–0.181819x11–0.011150x12 + 0.272292x13–0.011564x14–0.137367x15 + 0.360107x16 + 0.286721x17–0.675986x18–0.139855x19 + 0.198398x20–0.029304x21 + 0.103270x22 + 0.318297x23.

**Figure 4:**
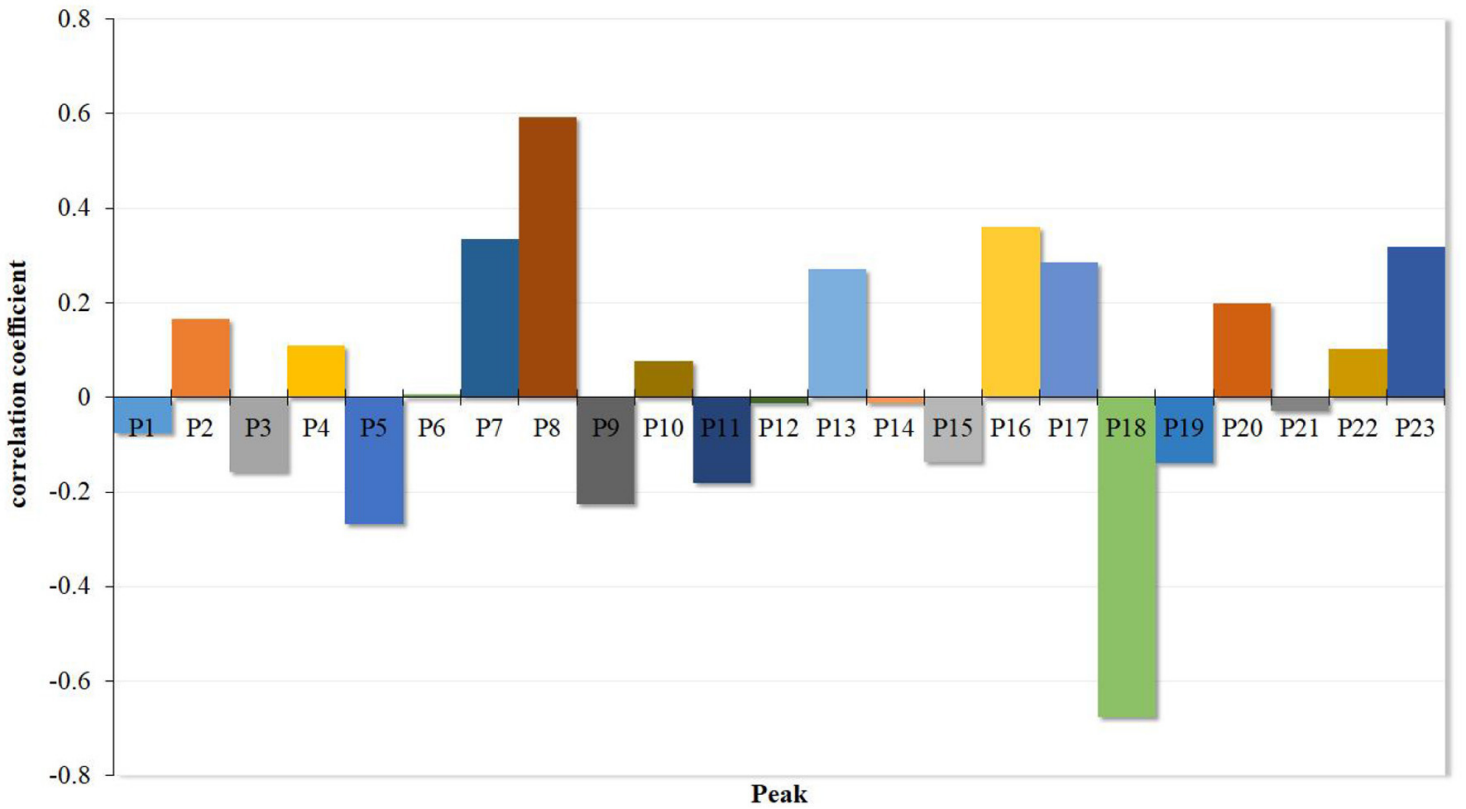
PLSR coefficient diagram of Poria.

The goodness of fit (*R*^2^ = 0.8954) was high enough to analyze the possible effect of the 23 common compounds on the α-glucosidase. All the regression coefficients of the 23 common compounds are represented in Figure 4. The results showed that **P2**, **P4**, **P7**, **P8**, **P10**, **P13**, **P16**, **P17**, **P20**, **P22**, and **P23** were positively effective in inhibiting α-glucosidase, while **P1**, **P3**, **P5**, **P9**, **P11**, **P12**, **P14**, **P15**, **P18**, **P19**, and **P21** were negatively effective, which means that increasing the proportion of the compounds with positive effects would enhance the α-glucosidase inhibitory ability of Poria.

### 3.2. Identification of the compounds with the activity of inhibiting α-glucosidase

#### 3.2.1. The effect of knock-out compounds and negative samples on α-glucosidase

**Figure 5:**
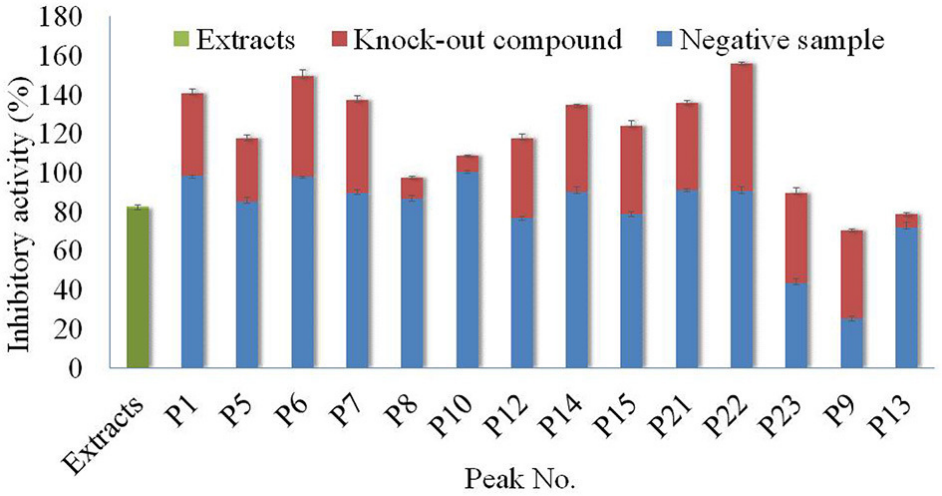
Analysis of antagonistic and synergistic effects between knock-out compounds of Poria extract and negative samples.

For further confirmation of the potential inhibition activity, the target compounds, **P1**, **P5**, **P6**, **P7**, **P8**, **P9**, **P10**, **P12**, **P13**, **P14**, **P15**, **P21**, **P22**, and **P23**, as well as corresponding negative samples, were used to test the inhibitory effect on α-glucosidase. However, these compounds with lower concentrations or poor resolution were not collected for further research. The results are shown in Figure 5.

In General, all the compounds and negative solutions showed some inhibitory effect on α-glucosidase. Among them, the target compounds including **P1**, **P5**, **P6**, **P7**, **P9, P12**, **P14**, **P15**, **P21, P22**, and **P23** exhibited strong inhibitory effect on α-glucosidase, while **P8**, **P10**, and **P13** showed weak inhibitory effect. Compared with the full extracts, the target compound **P9** and the corresponding negative solution would exhibit a lower effect on the α-glucosidase, while **P1**, **P5**, **P6**, **P7**, **P8**, **P10**, **P12**, **P14**, **P15**, **P21**, **P22** and their negative solutions showed stronger inhibitory effects, respectively. The sum effects of compounds **P23** and **P13** with their negative solutions were not significantly different from the full extracts. It can be observed that, for most compounds, separation was one effective way to alter their inhibitory effect on α-glucosidase (30).

Based on the comparison among these columns, it could be found that the inhibitory activity of each target compound and the negative solution was different. Taking P22 as an example, although the inhibitory activity of the full extracts was ~80%, the inhibitory activities of the compound and corresponding negative solution were almost 60 and 100%, respectively. The results also showed that separation was essential for using the specific function of the resource, or the function would not be demonstrated if the whole extract was used directly.

#### 3.2.2. Identification of target compounds

**Figure 6:**
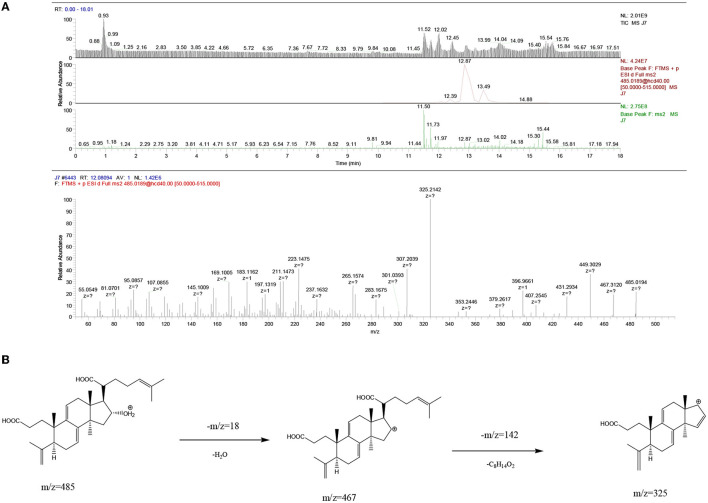
Mass spectra results of **P8** of Poria. **(A)** Positive ion mode; **(B)** the proposed fragmentation pathway of poricoic acid B.

The relative molecular masses of these target compounds were detected by positive ion modes under the mass spectrometry conditions of 2.2.6. Then, the possible molecular structure of the compounds was inferred from the secondary cleavage fragments by positive ion mode and from many relevant studies. After that, the compounds' structure was initially identified by the literature contrast of the polar order and UV absorption spectra with reported molecules (31–35). Finally, eight compounds, namely, **P8**, **P9**, **P10**, **P12**, **P13**, **P14**, **P21**, and **P22**, knocked out of Poria, were identified as follows:

The UPLC-MS^2^ result of **P8** (retention time of 12.08094 min) was shown in Figure 6, and a primary ion peak *m/z* 485.0189 [M + H]^+^ was yielded in positive ion mode, which revealed the presumed molecular weight of 484 and a possible molecular formula of C_30_H_44_O_5_. The secondary mass spectral fragmentation and cleavage pathway of the compound were shown in Figure 6B, indicating that it would be poricoic acid B.

By analogy, the UPLC-MS^2^ results of **P9**, **P10**, **P12**, **P13**, **P14**, **P21**, and **P22** were listed as Supplementary Figures S1–S7, respectively, and they were presumed to be dehydrotumulosic acid, poricoic acid A, polyporenic acid C, 3-epidehydrotumulosic acid, dehydropachymic acid, 3-O-Acetyl-16α-hydroxytrametenolic acid, and pachymic acid, respectively. The molecular details of them are listed in Table 4.

**Table 4:** Identification of chemical compounds in Poria.

**Peak No**.	**T_R_(min)**	**[M + H]^+^ (m/z)**	**Secondary ionic debris**	**Compound**	**Analytic diagram**
P8	12.08094	485.0194	467.3120, 325.2142	Poricoic acid B (C_30_H_44_O_5_)	Figure 6
P9	12.11050	485.0197	467.3505, 311.2349	Dehydrotumulosic acid (C_31_H_48_O_4_)	Supplementary Figure 1
P10	12.11223	499.4323	481.3296, 325.2140	Poricoic acid A (C_31_H_46_O_5_)	Supplementary Figure 2
P12	12.42158	483.0211	465.3350, 447.3237, 309.2191	Polyporenic acid C (C_31_H_46_O_4_)	Supplementary Figure 3
P13	12.60692	485.0191	467.3503, 449.3398, 311.2350	3-epidehydrotumulosic acid (C_31_H_48_O_4_)	Supplementary Figure 4
P14	12.67910	527.4883	509.3608, 353.2455, 449.3399	Dehydropachymic acid (C_33_H_50_O_5_)	Supplementary Figure 5
P21	12.74769	515.3712	497.3600, 479.3502, 437.3400, 293.2244	3-O-Acetyl-16α-hydroxytrametenolic acid (C_32_H_50_O_5_)	Supplementary Figure 6
P22	12.92147	529.3864	511.3762, 451.3554, 429.3864	Pachymic acid (C_33_H_52_O_5_)	Supplementary Figure 7

### 3.3. Molecular docking of target compounds with α-glucosidase

**Figure 7:**
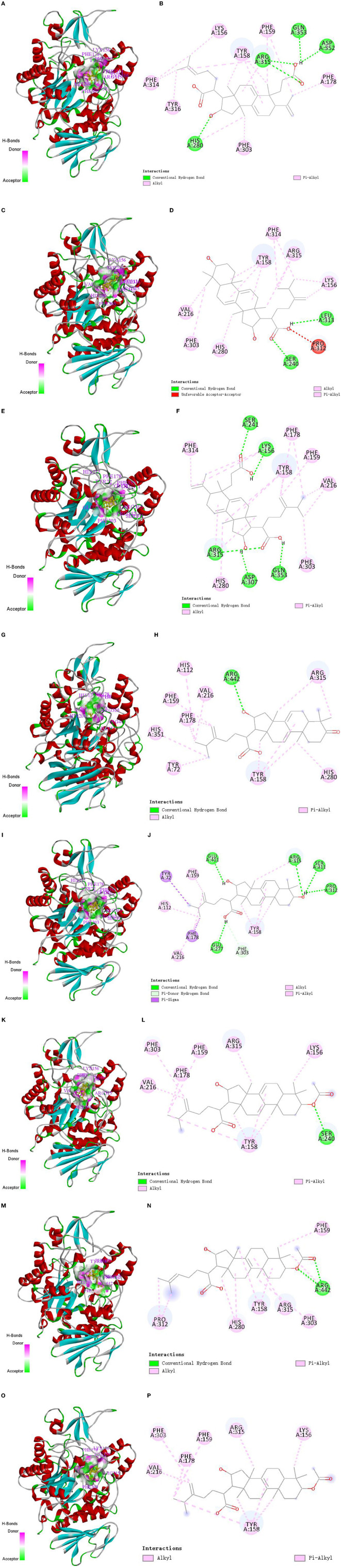
Molecular docking interaction of target compounds. **(A, B)** Poricoic acid B **(P8)**; **(C, D)** dehydrotumulosic acid **(P9)**; **(E, F)** poricoic acid A **(P10)**; **(G, H)** polyporenic acid C **(P12)**; **(I, J)** 3-epidehydrotumulosic acid **(P13)**; **(K, L)** dehydropachymic acid **(P14)**; **(M, N)** 3-O-Acetyl-16α-hydroxytrametenolic acid **(P21)**; **(O, P)** pachymic acid **(P22)**.

The molecular docking technique is a theoretical simulation method for predicting intermolecular binding sites and forces, which can provide further insight into the interaction mechanism of compounds with α-glucosidase (36, 37). In this study, molecular docking technique was used to simulate the binding sites and power of poricoic acid B (**P8**), dehydrotumulosic acid (**P9**), poricoic acid A (**P10**), polyporenic acid C (**P12**), 3-epidehydrotumulosic acid (**P13**), dehydropachymic acid (**P14**), 3-O-Acetyl-16α-hydroxytrametenolic acid (**P21**) and pachymic acid (**P22**) with α-glucosidase, and the result are shown in Figure 7.

The binding energy of poricoic acid B (**P8**), dehydrotumulosic acid (**P9**), poricoic acid A (**P10**), polyporenic acid C (**P12**), 3-epidehydrotumulosic acid (**P13**), dehydropachymic acid (**P14**), 3-O-Acetyl-16α-hydroxytrametenolic acid (**P21**), and pachymic acid (**P22**) with α-glucosidase were −8.9, −10.6, −9.2, −9.7, −9.9, −9.3, −9.3 and −9.3 kcal/mol, respectively. Their energy values were lower than −5.0 kcal/mol, which means that all eight compounds could bind with α-glucosidase steadily. It could be observed that the interaction between these compounds and α-glucosidase was mostly through alkyl and pi-alkyl with methyl groups and double bonds of C-17 side chain, A-ring, B-ring, or C-ring, followed by a conventional hydrogen bond with hydroxyl, carboxyl or acetoxy groups of C-3 and C-16. In addition, these compounds could also sometimes combine with the rings of α-glucosidase directly.

Specifically, if we take poricoic acid B (Figures 7A, B) as an example, poricoic acid B could form hydrogen bonds with amino acid residues of ARG: 315, ASP: 352, GLN: 353, and HIS: 280, as well as alkyl forces with PHE: 159, PHE: 178, PHE: 303, PHE: 314, TYR: 158, TYR: 316, and LYS: 156. The other compounds have similar interaction relationships with many amino acid residues of α-glucosidase. Therefore, it could be observed that the inhibitory ability of the compound on α-glucosidase was positively correlated with the ability of the compound to bind α-glucosidase, which indicated that the results of molecular docking were consistent with the results of compound activity screening, which might depend on the main forces of hydrogen bonds and alkyl bonds including Pi-Alkyl.

To elucidate interaction relationships clearly, the interaction relationships of the compounds with α-glucosidase were collected in Table 5. From the summarized results, it could be concluded that the main potential interaction sites of α-glucosidase were phenylalanine (PHE), tyrosine (TYR), arginine (ARG), valine (VAL), histidine (HIS), and others by the alkyl and conventional hydrogen bond, which was the possible working mechanism of Poria in inhibiting the activity of α-glucosidase.

**Table 5:** The interaction site of α-glucosidase with active compounds.

**Interaction site**	**PHE**	**TYR**	**VAL**	**ARG**	**HIS**	**LYS**	**PRO**	**GLN**	**ASP**	**SER**	**GLU**	**LEU**
Poricoic acid B	4 × Alk	2 × Alk		1 × Hyd	1^*^Hyd	1^*^Alk		1^*^Hyd	1^*^Hyd			
Dehydrotumulosic acid	2 × Alk	1 × Alk	1 × Alk	1 × Alk	1^*^Alk	1^*^Alk	1^*^UAA			1^*^Hyd		1^*^Hyd
Poricoic acid A	4 × Alk	1 × Alk	1 × Alk	1 × Hyd	1^*^Alk				1^*^Hyd	1^*^Hyd	1^*^Hyd	
Polyporenic acid C	2 × Alk	2 × Alk	1 × Alk	1 × Alk 1 × Hyd	3^*^Alk							
3-epidehydrotumulosic acid	1 × Alk 1 × PHB 1 × PSi	1 × Alk 1 × PSi	1 × Alk	1 × Hyd	1^*^Alk		1^*^Hyd			1^*^Hyd	2^*^Hyd	
Dehydropachymic acid	3 × Alk	1 × Alk	1 × Alk	1 × Alk		1^*^Alk				1^*^Hyd		
3-O-Acetyl-16α-hydroxytrametenolic acid	2 × Alk	1 × Alk		1 × Alk 1 × Hyd	1^*^Alk		1^*^Alk					
Pachymic acid	3 × Alk	1 × Alk	1 × Alk	1 × Alk		1^*^Alk						

Alk, Alkyl (including Pi-Alkyl); Hyd, conventional Hydrogen Bond; PSi, Pi-Sigma; UAA, unfavorable acceptor-acceptor; PHE, phenylalanine; TYR, tyrosine; VAL, valine; ARG, arginine; HIS, histidine; LYS, lysine; PRO, proline; GLN„ glutamine; ASP„ aspartic acid; SER, serine; GLU, glutamic acid; LEU, leucine.

## 4. Discussion

The study of the spectrum-effect relationship method has gradually attracted broad attention. Combined with the “component knock-out” technique, the spectrum-effect relationship method could discover the active substances from a complex system of samples such as Traditional Chinese Medicine (TCM), benefiting product development and quality control (38). The PLSR method was frequently used to establish a quantitative model of the spectrum-effect relationship to explore the information from the relationship (39, 40). In previous research, the spectrum-effect relationship method with PLSR successfully identified oxypeucedanin hydrate, imperatorin, cnidilin, isoimperatorin, byakangelicin, and bergapten from *Angelica dahurica*. The former four compounds were the main components with inhibitory effects on tyrosinase activity, while the latter two compounds had activating effects (21). In this study, 23 common spectrum peaks were matched from different batches of Poria samples according to HPLC fingerprints, and the PLSR method was used to establish the correlation between the peaks and α-glucosidase inhibitory activity. The results revealed that peaks **P2**, **P4**, **P6**, **P7**, **P8**, **P10**, **P13**, **P16**, **P17**, **P20**, **P22**, and **P23** were positively correlated with the inhibition of α-glucosidase activity, while peaks **P1**, **P3**, **P5**, **P9**, **P11**, **P12**, **P14**, **P15**, **P18**, **P19**, and **P21** were negatively correlated with the inhibitory ability.

The “component knock-out” technique was usually used to obtain the active target compounds from natural products and assist the spectrum-effect relationship analysis to test the structure and the activity of target compounds separately. For example, Liu et al. knocked out a series of compounds from the alcoholic extracts of *Begonia angustifolia* under the guidance of the spectrum-effect relationship and successfully found four compounds with a positive or negative effect on the tyrosinase, which were isochlorogenic acid B, isochlorogenic acid C, isochlorogenic acid A, and cymaroside (19). Shi et al. (41) successfully screened the main α-glucosidase inhibitor in pomegranate peel under the guidance of the spectrum-effect relationship and further confirmed that the inhibitor could significantly reduce postprandial blood sugar *in vivo*. The present study isolated 14 targeted compounds from Poria with potential inhibition effects using the “component knock-out” technique and tested their effect on the α-glucosidase activity, and the results showed that **P1**, **P5**, **P6**, **P7**, **P9, P12**, **P14**, **P15**, **P21**, **P22**, and **P23** had an inhibitory effect on α-glucosidase activity. Among them, the structures of eight compounds were determined using UPLC-MS^2^ and references as poricoic acid B, dehydrotumulosic acid, poricoic acid A, polyporenic acid C, 3- epidehydrotumulosic acid, dehydropachymic acid, 3-O-acetyl-16α-hydroxytrametenolic acid, and pachymic acid, respectively. According to many types of research, most of them, including dehydrotumulosic acid, polyporenic acid C, pachymic acid, dehydrotrametenolic acid, and dehydroeburicoic acid, could act as an insulin sensitizer in glucose tolerance tests and reduce hyperglycemia (42, 43). Xie et al. extracted poricoic acid A from *Sargassum pallidum* and also found the inhibitory effect of poricoic acid A on α-glucosidase (44). In addition, Xie also found that poricoic acid A could inhibit the formation of advanced glycation end products (AGEs) and fructosamine and have great potential as antiglycation inhibitors to treat diabetes (45). The mechanism in antidiabetics or the effect on α-glucosidase of the other bioactive compounds were not mentioned by scholars.

In recent years, the molecular docking technique has been widely used for the working mechanism of α-glucosidase inhibitors. Rahman et al. (46) studied the binding potential of 32 alkaloids with α-glucosidase using molecular docking techniques and found that nummular-R and vindoline had significant interaction with the enzyme; Ur-Rehman et al. (47) used molecular docking technique to study the connection of triterpenic acids isolated from Boswellia elongata with α-glucosidase and the results explored the inhibitory ability of these isolated compounds on α-glucosidase. In the research by Xie et al. (44), it was found that there were Four hydrogen bonds, alkyl and pi-alkyl, between poricoic acid A and α-glucosidase. This research identified 4 alkyl and four conventional hydrogen bonds between them. Similarly, the other seven compounds were docked with the residents of α-glucosidase, i.e., phenylalanine, tyrosine, arginine, valine, histidine, etc., via alkyl and hydrogen bonds, which were not reported before. The results showed that the alkyl and hydrogen bond between the compounds of Poria and the α-glucosidase lead to the inhibition of this enzyme, which would help to elucidate the mechanism of Poria treating diabetes.

## 5. Conclusion

In this research, the fingerprint of Poria was studied using HPLC analysis, and 23 common peaks were identified. Additionally, the inhibitory effect on the α-glucosidase of the 23 compounds of Poria was predicted using spectrum-effect relationship analysis and PLSR analysis. Combined with the “component knock-out” technique, LC-MS^2^, and molecular docking technique, 14 common compounds were knocked out, and the molecule structure of eight active compounds was identified for further analysis. Based on the analysis, it was concluded that Poricoic acid B, dehydrotumulosic acid, poricoic acid A, polyporenic acid C, 3-epidehydrotumulosic acid, dehydropachymic acid, 3-O-acetyl-16α-hydroxytrametenolic acid, and pachymic acid exhibited a significantly inhibitory effect on the α-glucosidase, and their inhibitory effect might be related to the interaction with α-glucosidase mainly through alkyl and hydrogen bonds. These results, including the HPLC fingerprint and bioactive compound of Poria, can be used for the quality evaluation and control of Poria and the development of effective medicines for the treatment of diabetes.

## Data availability statement

The original contributions presented in the study are included in the article/Supplementary material, further inquiries can be directed to the corresponding authors.

## Author contributions

CM and JL designed the study and performed experiments, writing, and original draft preparation. MR and QW analyzed the summarized data and verified it. CL and XX contributed to the data acquisition, project administration, and critically reviewed the manuscript. ZL provided resources and funding and reviewed the manuscript. All authors contributed to the article and approved the submitted version.
