# Supplementary material for: Rapid identification of α-glucosidase inhibitors from Poria using spectrum-effect, component knock-out, and molecular docking technique
Source: Front Nutr. 2023 Aug 10;10:1089829. doi: 10.3389/fnut.2023.1089829 (PMC10448901; doi:10.3389/fnut.2023.1089829)
Supplement: Supplementary file 1 [file Data_Sheet_1.docx]

**Supplementary Figures**

(a)

(b)

Fig. S1 Mass spectra results of P9 of Poria

(a. Positive ion mode; b. The proposed fragmentation pathway of dehydrotumulosic acid)

(a)

(b)

Fig. S2 Mass spectra results of P10 of Poria

(a. Positive ion mode; b. the proposed fragmentation pathway of poricoic acid A)

(a)

(b)

Fig. S3 Mass spectra results of P12 of Poria

(a. Positive ion mode; b. the proposed fragmentation pathway of polyporenic acid C)

(a)

(b)

Fig. S4 Mass spectra results of P13 of Poria

(a. Positive ion mode; b. the proposed fragmentation pathway of 3-epidehydrotumulosic acid)

(a)

(b)

Fig. S5 Mass spectra results of P14 of Poria

(a. Positive ion mode; b. the proposed fragmentation pathway of dehydropachymic acid)

(a)

(b)

Fig. S6 Mass spectra results of P21 of Poria

(a. Positive ion mode; b. the proposed fragmentation pathway of 3-O-Acetyl-16α-hydroxytrametenolic acid)

(a)

(b)

Fig. S7 Mass spectra results of P22 of Poria.

(a. Positive ion mode; b. the proposed fragmentation pathway of pachymic acid)
